# Mandibular Cemento‑ossifying Fibroma

**DOI:** 10.5334/jbsr.3872

**Published:** 2025-03-28

**Authors:** Michael Matthys, Filip M. Vanhoenacker

**Affiliations:** 1 AZ Sint‑Maarten, Mechelen, and UZ Brussel, Brussel, Belgium; 2 AZ Sint‑Maarten, Mechelen, and University (Hospital), Antwerp/Ghent, Belgium

**Keywords:** Cemento‑ossifying fibroma, Mandible, Cone beam CT, MRI

## Abstract

*Teaching point:* Cemento‑ossifying fibroma is a benign tumor of the jaws with a mixed radiolucent–radiopaque appearance and causes root resorption and bone expansion.

## Case History

A 44‑year‑old female presented with submucosal swelling at the right lower lip.

**Figure 1:**
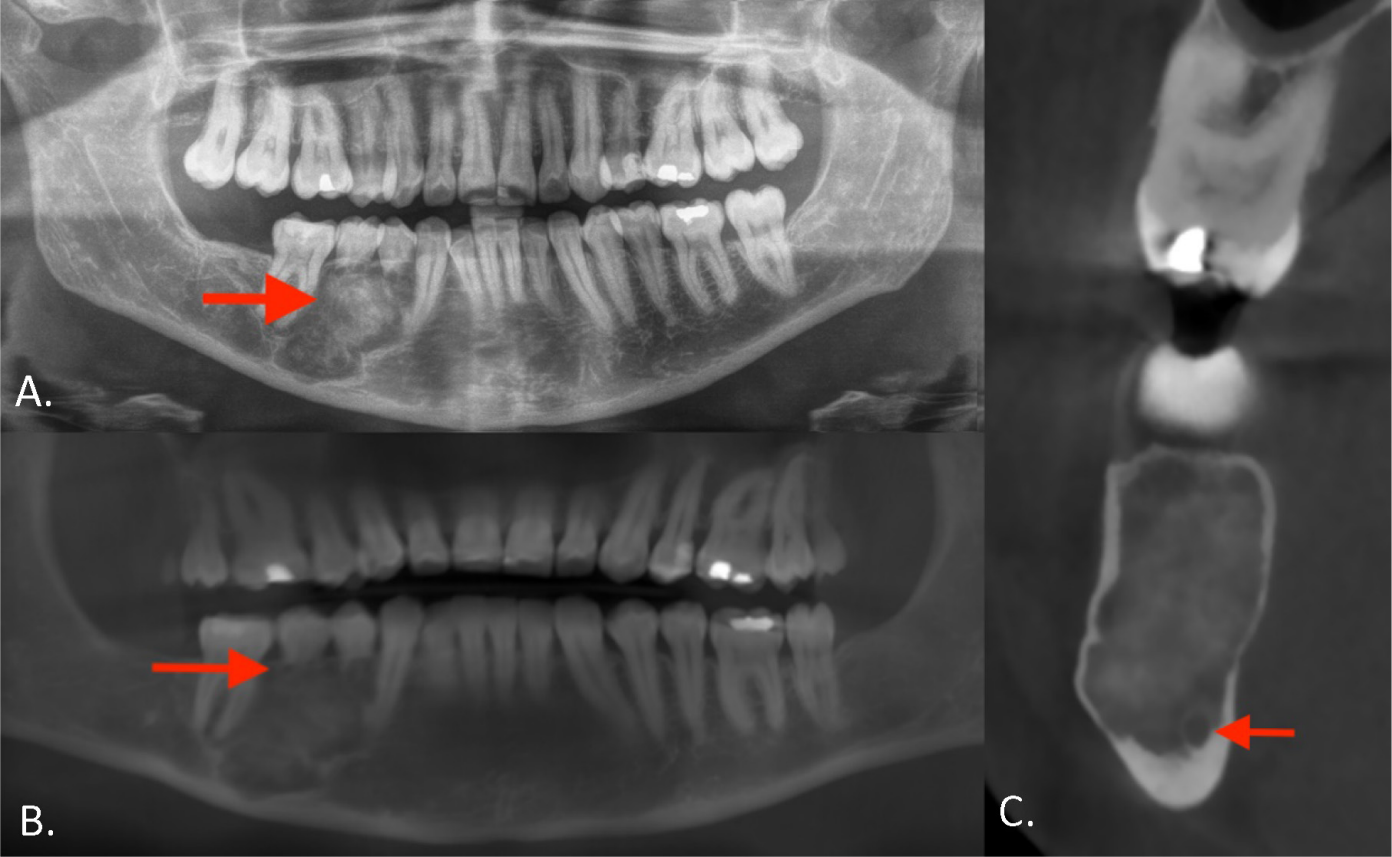
**(A)** Panoramic radiograph shows a rounded lesion with a radiopaque core and surrounding radiolucent halo. **(B)** Coronal reformatted CBCT views confirm the mixed radiolucent‑radiopaque lesion with ground‑glass appearance of the central part. **(C)** Sagittal reformatted CBCT view shows a cortical thinning of the buccal and lingual aspect of the mandible with a sparing of mandibular canal (arrow).

Panoramic radiograph showed a rounded lesion consisting of a radiopaque core surrounded by a radiolucent halo (Figure 1A, arrow).

Cone beam computed tomography (CBCT) confirmed a mixed radiolucent–radiopaque lesion with ground‑glass appearance of the central part (arrows). There was extensive root resorption of teeth 44 and 45 and partial resorption of teeth 43 and 46 (Figure 1B, arrow). Cortical thinning of the buccal and lingual aspect of the mandible was also seen. The lesion extended toward the mandibular canal with sparing of its cortical delineation (Figure 1C, arrow).

**Figure 2:**
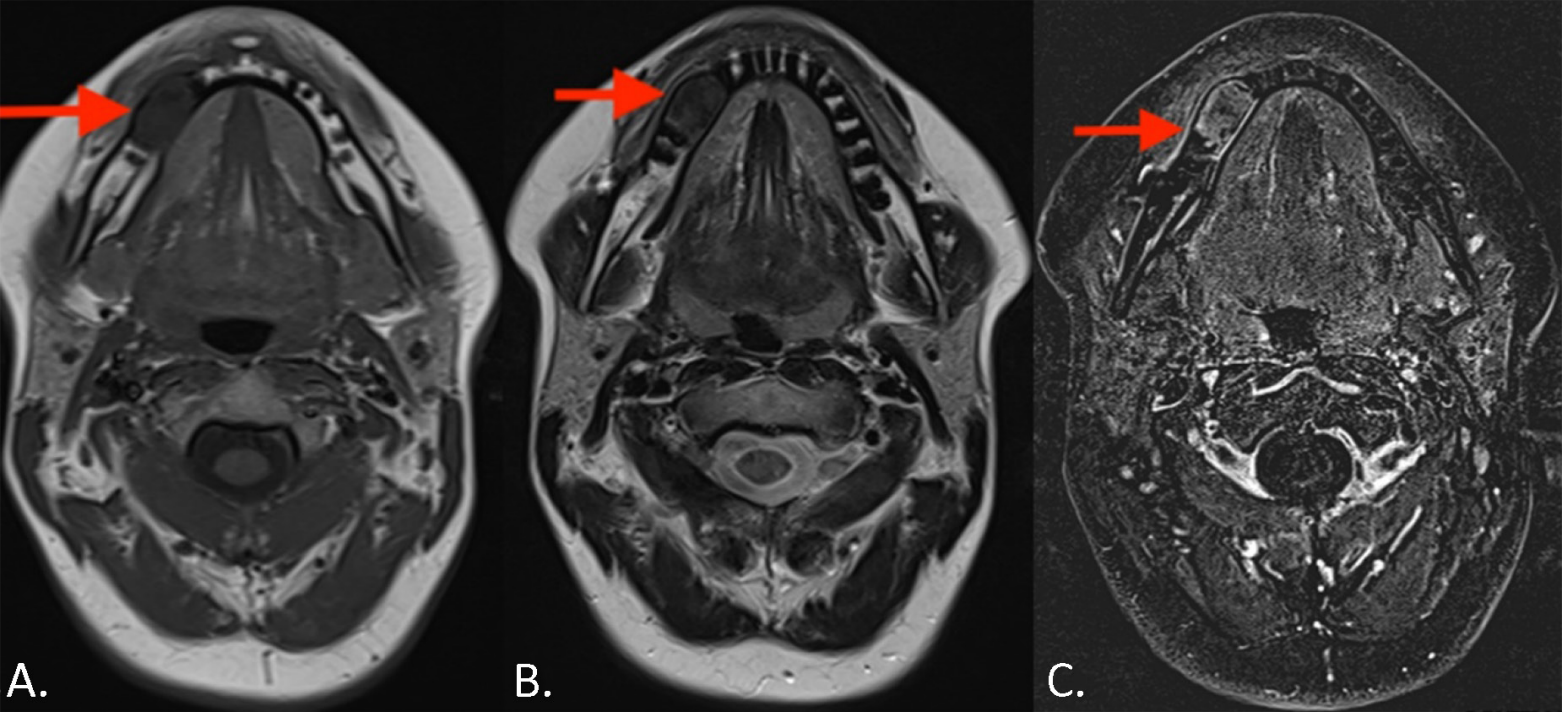
**(A‑B)** The lesion in hypointense on both T1‑ and T2‑ WI. **(C)** Axial subtraction image after contrast administration shows heterogeneous predominant peripheral enhancement.

Magnetic resonance imaging (MRI) showed a well‑delineated lesion at the roots of teeth 43–46. The lesion was hypointense on T1‑weighted images (WI) (Figure 2A, arrow) and T2‑WI (Figure 2B, arrow). There was no soft tissue extension. Diffusion restriction was absent (not shown). After intravenous gadolinium contrast administration (Figure 2C, arrow), there was slight heterogeneous enhancement predominantly at the periphery. Based on the imaging findings, the preferred diagnosis was cemento‑ossifying fibroma (COF), which was confirmed on histopathology.

## Comment

COF is a benign fibro‑osseous non‑odontogenic tumor of the jaws originating from mesenchymal cells within the periodontal ligament. It predominantly affects women in their 30s and 40s.

It usually grows slowly and is therefore often detected incidentally in asymptomatic patients. Larger lesions may lead to facial asymmetry, discomfort, and dental displacement.

The lesion is well‑defined and mixed radiolucent–radiopaque on (panoramic) radiographs and (cone beam) computed tomography [1]. Its fibrous matrix containing cement‑like calcifications and ossifications results in a ground‑glass appearance of the opaque part of the lesion. Longstanding lesions may become more radiopaque owing to progressive matrix mineralization.

A cemento‑ossifying fibroma (COF) is hypointense on both T1‑ and T2‑weighted MRI owing to its dense, collagen‑rich fibrous matrix and mineralized components [1]. The low‑fat content also contributes to its hypointensity on T1‑WI. Contrast enhancement is variable after intravenous gadolinium contrast administration.

Treatment consists of surgical excision. Recurrence following complete removal is rare.

The radiological differential diagnosis of cemento‑ossifying fibroma (COF) includes fibrous dysplasia (FD), odontoma, cementoblastoma, osteosarcoma, and chondrosarcoma. FD also has a “ground‑glass” appearance but may displace the mandibular canal in any direction, whereas a large COF tends to displace the mandibular canal inferiorly. Odontomas are highly radiopaque and associated with unerupted teeth, while cementoblastomas typically fuse with the roots of the adjacent teeth. Osteosarcomas and chondrosarcomas have aggressive imaging features with an osteoid and chondroid matrixmetric, respectively. A chondroid matrix is typically of high signal intensity on T2‑WI.
